# Risk of Severe Knee and Hip Osteoarthritis in Relation to Level of Physical Exercise: A Prospective Cohort Study of Long-Distance Skiers in Sweden

**DOI:** 10.1371/journal.pone.0018339

**Published:** 2011-03-30

**Authors:** Karl Michaëlsson, Liisa Byberg, Anders Ahlbom, Håkan Melhus, Bahman Y. Farahmand

**Affiliations:** 1 Department of Surgical Sciences, Section of Orthopaedics, Uppsala University, Uppsala, Sweden; 2 Uppsala Clinical Research Center, Uppsala University, Uppsala, Sweden; 3 The Institute of Environmental Medicine, Karolinska Institutet, Stockholm, Sweden; 4 Department of Medical Sciences, Section of Clinical Pharmacology, Uppsala University, Uppsala, Sweden; Central Institute of Educational Technology, Canada

## Abstract

**Background:**

To complete long-distance ski races, regular physical exercise is required. This includes not only cross-country skiing but also endurance exercise during the snow-free seasons. The aim of this study was to determine whether the level of physical exercise is associated with future risk of severe osteoarthritis independent of previous diseases and injuries.

**Methodology/Principal Findings:**

We used a cohort that consisted of 48 574 men and 5 409 women who participated in the 90 km ski race Vasaloppet at least once between 1989 and 1998. Number of performed races and finishing time were used as estimates of exercise level. By matching to the National Patient Register we identified participants with severe osteoarthritis, defined as arthroplasty of knee or hip due to osteoarthritis. With an average follow-up of 10 years, we identified 528 men and 42 women with incident osteoarthritis. The crude rate was 1.1/1000 person-years for men and 0.8/1000 person-years for women. Compared with racing once, participation in ≥5 races was associated with a 70% higher rate of osteoarthritis (multivariable-adjusted hazard ratio (HR) 1.72, 95% confidence interval (CI) 1.33 to 2.22). The association was dose-dependent with an adjusted HR of 1.09, 95% CI 1.05 to 1.13 for each completed race. A faster finishing time, in comparison with a slow finishing time, was also associated with an increased rate (adjusted HR 1.51, 95% CI 1.14 to 2.01). Contrasting those with 5 or more ski races and a fast finish time to those who only participated once with a slow finish time, the adjusted HR of osteoarthritis was 2.73, 95% CI 1.78 to 4.18.

**Conclusions/Significance:**

Participants with multiple and fast races have an increased risk of subsequent arthroplasty of knee and hip due to osteoarthritis, suggesting that intensive exercise may increase the risk.

## Introduction

Undoubtedly, participation in sports and exercise has a positive effect on health in most people [1]–[4]. Evidence shows that moderate habitual exercise at middle and old age does not increase the risk of developing osteoarthritis [5]. Nevertheless, it is well known that trauma or injury to the knee is associated with future osteoarthritis [6], [7] and that vigorous sports activities increase the risk of acute joint injuries [7]. An important question is whether prolonged intensive exercise may lead to osteoarthritis [5]. Animal models indicate that moderate weight and non-weight bearing physical activity do lead to either reversible pathologic changes in cartilage composition or to osteoarthritis in some [8]–[10] but not all studies [11]. Research in humans has also been inconsistent: some studies indicate a higher risk of knee or hip osteoarthritis after previous sport participation, whereas others could not show this association [5], [12]–[17]. We sought to delineate how the level of physical exercise independent of previous injuries affects the future risk of severe osteoarthritis in a large cohort of long-distance skiers.

## Methods

### Ethics statement

The study protocol was approved by the Regional Ethical Review Board, Karolinska Institutet, Stockholm, Sweden.

### Study participants

The present cohort study is based on finishing Swedish participants in the 90 km long-distance cross-country ski marathon race Vasaloppet in Sweden (www.vasaloppet.se) which is held annually the first Sunday in March. We restricted the study base to participants of the main classical race. Vasaloppet is the oldest, longest, and largest cross-country ski race in the world. The first Vasaloppet was run in 1922 with 119 participants. Nowadays, more than 15 000 men and women annually take part in the main long race (www.vasaloppet.se). In the period 1924 to 1980, women were not allowed to participate in the race. Even if the participating number of women after the restriction period gradually increased during the 1980s, they are still in 2010 underrepresented (1∶10 of the participants).

The vast majority of the participants are not competing on the elite levels but the best competitors have an extremely high physical capacity. There is also a close relationship between average speed or ranking in ski races and maximum aerobic power (VO_2max_), r^2^ values being in the range of 0.9 [18]. Winners of the Vasaloppet who finish the race in about 4 h have a VO_2max_ of 80 ml min^−1^ kg^−1^ or higher [19], whereas skiers who finish in 10–13 h, i.e., the slowest speed in the race, have VO_2max_ of at least 45 ml min^−1^ kg^−1^ or higher [20] compared with an average VO_2max_ of 36 and 33 ml min^−1^ kg^−1^ amongst men and women in the general population [21]. We have previously shown that participants in Vasaloppet report a higher leisure physical activity, lower frequency of smoking, obesity, high fat consumption, and physical and mental illness [4]. Accordingly, they have a reduced standardized mortality ratio, and the reduction is most pronounced for subjects who participated in several races and for deaths due to cardiovascular disease [4].

The Vasaloppet office provided information on all Swedish citizens (49 117 men and 5 443 women) that completed any of the 90 km Vasaloppet races from 1989–1998, including information on year of race and finishing time, as well as each participant's 10-digit Swedish identification number. The race in 1990 was cancelled because of thawing.

The records of all participants were matched to the National Patient Registry for identification of diagnoses through December 31, 2005. Information on date of death was attained from the Swedish Causes of Death Register. The cohort was also linked to the Swedish National Population Registry to collect information on emigration.

We excluded 463 individuals (439 men and 24 women) from the study base because prior to study entry (i.e., the last race) they had had a diagnosis of osteoarthritis (n = 225, with or without surgical procedure done), or a diagnosis of inflammatory joint disease, including rheumatoid arthritis (n = 235), or both (n = 3). Another 114 skiers with a diagnosis of secondary osteoarthritis were excluded from further analysis (104 men, 10 women). The remaining 53 983 participants formed our study base.

### Exposures

Since those who participated in several races tended to ski faster races (Spearman rank correlation 0.45, p<0.001) and improvements in VO_2max_ are directly related to intensity, duration, and frequency of training [22], the number of successful participations in Vasaloppet and the finishing times in the period from 1989 through 1998, as continuous variables and in categories, were used as estimates of level of physical exercise [4]. Number of races and finishing time before 1989 or after 1998 were not considered in the exposure assessment. Nonetheless, it is important to emphasize that this exposure to exercise is not restricted to skiing, e.g. long-distance running and roller skiing are common activities during the preparation for the race when it is not possible to ski. The skiing season normally lasts 3 months. In our previous study we showed that regular strenuous leisure exercise was typically performed by 21% and strenuous exercise by 58% of the participants in Vasaloppet as compared with 4 and 25%, respectively, in the general population [4] Sixty-four percent of the participants had at least 4 h/week of regular training while only 17% of the population exercised 1.5 h or more per week.

Finishing time was, on average, about 8 h (range 4–13 h). As skiing conditions vary from year to year, we expressed finishing time relative to the winner time as previously defined [4] in four categories as 100–160, 161–200, 201–240 and 241% or more than the winner's time (year- and sex-specific). For those who had participated in several races, the lowest relative finishing time was used as exposure.

### Outcomes

Information on knee and hip arthroplasty for osteoarthritis after the last race of the participant through 2005 were based on record linkage with the National Patient Registry. Severe knee and hip osteoarthritis were defined as previously described [23], i.e., as a first knee arthroplasty or high tibial osteotomy (procedures coded 8424, 8423, 8428, 8010, 8199 or NGB09, NGB19, NGB29, NGB39, NGB49, NGB99 and NGK59) in combination with a contemporaneous diagnosis of osteoarthritis (715 or M17 according to the International Classification of Disease (ICD) 9 and ICD-10, respectively). Only the first event was counted for patients with more than one knee arthroplasty. Severe hip osteoarthritis was defined as a first hip arthroplasty (procedures coded 8414, 8010, NFB09, NFB19, NFB29, NFB39, NFB49, NFB99) in combination with a contemporaneous diagnosis of hip osteoarthritis (715 or M16 according to ICD-9 and ICD-10, respectively). Patients with more than one hip arthroplasty were only counted once. The annually updated National Patient Registry covers all inpatient care in Sweden since 1987. and complete individual matching to the register is enabled by the personal registration number provided to all Swedish citizens [24]. It has been validated against the national Swedish arthroplasty registers and was estimated to include at least 95% of primary knee and hip arthroplasties [25].

### Additional information

In order to assess and control for diseases and injuries before the last race and during follow-up, infectious arthritis; diabetes mellitus; other endocrine disorders; fractures; contusions, distortions, dislocations and other injury diagnoses were determined from the National Patient Registry to the end of follow-up by ICD-7 through ICD-10 codes, depending on the period of diagnosis ascertainment. The frequency of injuries associated with cross-country skiing is, however, low compared to most other athletic activities [26].

The cohort was linked to the censuses of 1960, 1970, 1980, and 1990 to retrieve information on occupation, educational level, and household income. Occupation was grouped into four categories (blue-collar, lower-middle white-collar, high white-collar, and entrepreneur). Education was grouped into three categories: low (elementary school), medium (secondary school), and high (university).

### Statistical analyses

The number of person-years was ascertained individually for all participants in the cohort. The date of entry to the study was the date of the last race during the years 1989–1998. Date of the completion of follow-up was the first date of osteoarthritis diagnosis, date of death, date of emigration, or the end of follow-up on 31 December 2005, whichever came first.

Cox's proportional hazards regression models were used to assess hazard ratios with 95% confidence intervals for primary in-hospital treated osteoarthritis. Only the first event of each osteoarthritis category (hip or knee osteoarthritis) was considered for patients with more than one hospital stay.

We modelled osteoarthritis risk by number of successful races or by finishing time. The number of competitions was analyzed as a continuous variable or in four predefined [4] groups (one, two, three to four, and five or more races). Finishing time relative to the winning time was analyzed also in four predefined [4] categories. Similar results were obtained if we used average finishing time of the successful races instead of the relative fastest finishing time for those who participated in several races. We additionally considered the combined influence of successful races and finishing time on the association with future risk of osteoarthritis by contrasting those with several performed races (five or more races) and a fast finishing time (fastest category) to participants with only one finished, slow pace race.

Two models were fitted. The first one included age (continuous), sex, socioeconomic status by occupation and education as covariates. We extended this model to adjust our estimates for separate marker variables (all dichotomous) for concomitant disorders that can influence the risk of osteoarthritis and exercise level: infectious arthritis, diabetes mellitus, other endocrine disorders, fractures, dislocations, distortions, contusions, any other acute injury, and any other chronic injury.

## Results

The characteristics of participants are shown in Table 1. During a total of 526 472 person-years of follow-up with an average follow-up of 10 years (range 0–16 years) per individual, 528 men and 42 women were treated with arthroplasty of the knee or hip due to osteoarthritis after their last ski race. As expected, the incidence of severe osteoarthritis increased with increasing age and with the crude occurrence of previous injuries. The distribution of participants and number of cases with incident severe osteoarthritis of the hip and knee during follow-up by year of the last race is provided in the Table S1. Most participants had their last race in the later part of the inclusion period.

**Table 1:** Characteristics with incidence of osteoarthritis (OA) at the hip or the knee among participants in the cross-country ski race Vasaloppet.

	N participants	N cases with OA	Incidence OA per 1000 person-years
**Total**	53983	570	1.08
**Age (years)**			
**15**–**29**	15248	18	0.12
**30**–**39**	14589	56	0.39
**40–49**	12139	130	1.08
**50–59**	8997	221	2.66
**≥60**	3010	145	5.39
*Mean age (SD)*	*38.8 (12.3)*		
**Successful races**			
1	28012	214	0.73
2	10052	106	1.10
3–4	8648	105	1.35
≥5	7271	145	2.46
**Finish time (% of winner time)**			
100–160	9631	105	1.17
161–200	17504	206	1.22
201–240	16078	170	1.07
≥241	10770	89	0.81
**Education**			
Low	11511	189	1.69
Medium	23011	184	0.81
High	16344	173	1.08
**Occupation**			
Blue-collar	18449	210	1.15
Low-middle white-collar	18802	207	1.12
High white-collar	7529	98	1.34
Entrepreneur	2830	47	1.68
**Medical conditions**			
Dislocation	529	15	2.94
Distortion	1723	30	1.81
Contusion	861	17	2.04
Neurological disorder	118	2	1.96
Diabetes mellitus	169	2	1.22
Endocrine diseases	468	7	1.59
Fractures	3445	41	1.24
Chronic injuries	4049	93	2.49
Acute injuries	5940	93	1.66

Those who repeatedly had participated in the competition had a higher risk of osteoarthritis. Participants who had taken part in several races displayed an increased rate of hip or knee osteoarthritis with a 9% (hazard ratio 1.09, 95% CI 1.05 to 1.13) increase per successful race (Table 2). This estimate was only marginally influenced if co-morbidities, including previous injuries, were accounted for in the multivariable model (hazard ratio 1.08 per race, 95% CI 1.04 to 1.13). Using the same model and restraining the analysis to those with a last participation in the race in either of the last two years of the inclusion period (1997–1998), provided a similar estimate (hazard ratio 1.10 per race, 95% CI 1.04–1.16). In addition, restriction of the cohort to only those without an injury diagnosis still revealed a higher risk of osteoarthritis (hazard ratio 1.11 per race, 95% CI 1.06 to 1.16). Those who had participated in 5 or more races had a 72% (hazard ratio 1.72, 95% CI 1.33 to 2.22) higher rate of having a diagnosis of osteoarthritis of the knee or hip compared with participants who had taken part in only one race event.

**Table 2:** Hazard ratios (HRs) and 95% confidence intervals (CIs) of osteoarthritis associated with number of successful races.

	HipN = 356 cases		KneeN = 227 cases		Hip or kneeN = 570 cases	
	HR^a^ (95% CI)	HR^b^ (95% CI)	HR^a^ (95% CI)	HR^b^ (95% CI)	HR^a^ (95% CI)	HR^b^ (95% CI)
**Successful races**						
**1**	1.00	1.00	1.00	1.00	1.00	1.00
**2**	1.25 (0.91–1.69)	1.24 (0.91–1.69)	1.41 (0.97–2.05)	1.38 (0.95–2.00)	1.33 (1.05–1.69)	1.32 (1.04–1.68)
**3–4**	1.34 (0.98–1.83)	1.31 (0.96–1.80)	1.35 (0.90–2.02)	1.31 (0.87–1.96)	1.33 (1.04–1.72)	1.31 (1.01–1.68)
**≥5**	1.59 (1.15–2.20)	1.59 (1.15–2.20)	1.83 (1.21–2.76)	1.73 (1.14–2.63)	1.72 (1.33–2.22)	1.68 (1.30–2.17)
**Per race**	1.08 (1.03–1.14)	1.08 (1.03–1.14)	1.09 (1.02–1.16)	1.08 (1.01–1.15)	1.09 (1.05–1.13)	1.08 (1.04–1.13)

a Adjusted for age (continuous), gender, education (low, medium, high) and occupation (blue-collar, lower-middle white-collar, high white-collar and entrepreneur).

b Adjusted for all variables in the model ^a^ and, in addition, the following diagnoses: any fracture, any acute injury, any chronic injury, any distortion, any dislocation, any contusion, any neurologic disease, infectious arthritis, diabetes mellitus, and any other endocrine disorder.

The individual's race pace was also of importance (Table 3). For every category of a faster finishing time, we found a 13% (hazard ratio 1.13, 95% CI 1.03 to 1.23) increased rate of osteoarthritis of the knee or hip. Those who had a finishing time close to the winner (within 60% of his time) had a 50% (hazard ratio 1.51, 95% CI 1.14 to 2.01) increased rate of osteoarthritis of the knee or hip compared with the slowest competitors.

**Table 3:** Hazard ratios (HRs) and 95% confidence intervals (CIs) of osteoarthritis associated with finishing time.

	HipN = 356 cases		KneeN = 227 cases		Hip or kneeN = 570 cases	
	HR^a^ (95% CI)	HR^b^ (95% CI)	HR^a^ (95% CI)	HR^b^ (95% CI)	HR^a^ (95% CI)	HR^b^ (95% CI)
**Finishing time** **(% of winner time)**						
**≥241**	1.00	1.00	1.00	1.00	1.00	1.00
**201–240**	1.20 (0.87–1.67)	1.21 (0.87–1.68)	0.99 (0.67–1.48)	1.00 (0.67–1.48)	1.13 (0.87–1.46)	1.13 (0.88–1.47)
**161–200**	1.26 (0.92–1.74)	1.25 (0.91–1.73)	0.99 (0.67–1.46)	0.97 (0.66–1.43)	1.17 (0.91–1.50)	1.15 (0.90–1.48)
**100–160**	1.54 (1.06–2.24)	1.53 (1.05–2.23)	1.41 (0.91–2.16)	1.34(0.87–2.07)	1.51 (1.14–2.01)	1.48 (1.11–1.97)
**Per category**	1.14 (1.01–1.27)	1.13 (1.01–1.26)	1.10 (0.96–1.26)	1.08 (0.94–1.24)	1.13 (1.03–1.23)	1.12 (1.02–1.22)

a Adjusted for age (continuous), gender, education (low, medium, high) and occupation (blue-collar, lower-middle white-collar, high white-collar and entrepreneur).

b Adjusted for all variables in the model ^a^ and, in addition, the following diagnoses: any fracture, any acute injury, any chronic injury, any distortion, any dislocation, any contusion, any neurologic disease, infectious arthritis, diabetes mellitus, and any other endocrine disorder.

We finally contrasted those with 5 or more ski races and a fast finishing time to those who only participated once and did so with a slow finishing time (Table 4). The former intensive exercise category (5 or more ski races combined with a fast time), had a nearly three-fold increased rate of osteoarthritis of the knee or hip compared with those in the second category (those who participated only once with a slow finishing time), also after controlling for previous injuries. Notably, the hazard ratio for hip osteoarthritis alone was 3.15, 95% CI 1.79 to 5.52. Moreover, we found no evidence that those in the intensive exercise category had had more previous injuries (odds ratio by multivariable logistic regression 1.01, 95% CI 0.88 to 1.16).

**Table 4:** Hazard ratios (HRs) and 95% confidence intervals (CIs) of osteoarthritis associated with combination of finishing time and number of races.

		HipN = 72 cases		KneeN = 49 cases		Hip or kneeN = 119 cases	
		HR^a^ (95% CI)	HR^b^ (95% CI)	HR^a^ (95% CI)	HR^b^ (95% CI)	HR^a^ (95% CI)	HR^b^ (95% CI)
**Finish time** **(% of winner time)**	**N races**						
**≥241**	**1**	1.00	1.00	1.00	1.00	1.00	1.00
**100–160**	**≥5**	3.11 (1.79 to 5.40)	3.18 (1.81 to 5.60)	1.95 (1.02 to 3.74)	1.96 (1.02 to 3.77)	2.73 (1.78 to 4.18)	2.74 (1.78 to 4.23)

a Adjusted for age (continuous), gender, education (low, medium, high) and occupation (blue-collar, lower-middle white-collar, high white-collar and entrepreneur).

b Adjusted for all variables in the model ^a^ and, in addition, the following diagnoses: any fracture, any acute injury, any chronic injury, any distortion, any dislocation, any contusion, any neurologic disease, infectious arthritis, diabetes mellitus, and any other endocrine disorder.

**Figure 1:**
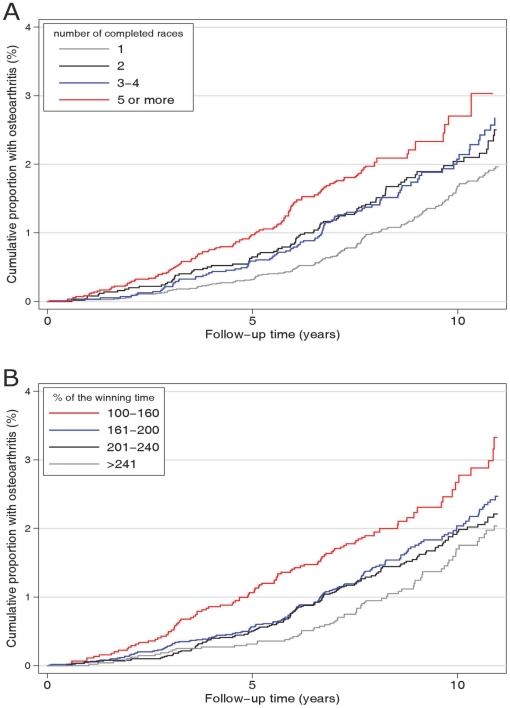
Age- and sex-adjusted survival curves of osteoarthritis by successful races (panel A) and finishing time (panel B).

Survival curves of knee or hip osteoarthritis by number of successful races and categories of finishing time are displayed in Figure 1. The survival curves have been adjusted for sex distribution (male to female ratio 10∶1) and median age at study entry of cases (53 years). The 10-year probability to receive surgery for osteoarthritis was approximately 3% in participants with 5 or more successful ski races and in those with fastest average ski speed. The corresponding number for those in the contrasting categories with only one race performed or a low skiing speed was about 2%.

## Discussion

Our data show a relation between the number of races completed and the time to completion and the risk of subsequent arthroplasty of the knee or hip due to osteoarthritis, suggesting that the intensive exercise required to most successfully complete the long-distance race Vasaloppet may be associated with an increased risk of osteoarthritis. The associations were independent of previous injuries identified from registry data.

There are no previous prospective investigations of this size that have examined intensive exercise and future risk of osteoarthritis. In a 9-year follow-up in 28 regular runners compared with matched controls, no radiological evidence of differences in osteophyte progression or narrowing of joint space by regular radiographs of the hips and knees was found [27]. In another small study with nine male triathletes and nine controls quantitative magnetic resonance imaging (MRI) was used for analysis of knee joint cartilage thickness [28]. A tendency of diminished cartilage volume was found at weight bearing sites in the athletes.

Prospective studies of individuals with less intensive physical activity level have presented conflicting results [12]. The most recent and thoroughly performed study from the Framingham Offspring cohort concluded that there was no association between regular physical activity level and development of knee osteoarthritis over a 9-year follow-up in 1 279 men and women with an average age of 53 years at baseline [29]. Few of these men and women reported performing intensive exercise. Earlier investigations based on the original Framingham study found that those who reported high physical activity had a higher future risk of radiographic signs of osteoarthritis [30], [31]. Men investigated at the Cooper Clinic (Dallas, TX, US) who were younger than 50 years of age and who participated in high physical activity had an increased risk of self-reported but not physician-diagnosed osteoarthritis of the hip and knee [32]. When controlling for previous injuries in a later follow-up in the same cohort, no evident risk increase was noted [33]. No association between physical activity level and joint space narrowing or osteophyte occurrence was observed in the Chingford study [34]. In an Australian longitudinal study low physical activity conferred a reduced risk for radiological signs of knee osteoarthritis but only for the physical activity level during 20–29 years of age and not in the four other age categories [35]. Radiographic signs of knee osteoarthritis were associated with regular sports activities in a British cohort study of 354 participants but previous injuries were not adjusted for and progression of osteoarthritis was not influenced by regular sports participation [36].

Hence, self-reported physical activity has been used in most cohort studies. Some self-report measures have been validated and are thus feasible for use in large studies but the method is prone to misclassification and might not capture the type and true intensity of the physical exercise it is aimed to measure, leading to attenuated risk estimates. In addition, failure to account for previous injuries in the analysis or design of the study might overestimate the influence of high physical activity because these factors can act together. We have used the number of completed races and finishing time in a long-distance skiing competition as more objective estimates of physical exercise. Winners of the Vasaloppet belong to the group of world-class cross-country skiers. However, the vast majority of the participants do not compete on the elite levels. But even the slowest competitors in the Vasaloppet have a higher physical capacity and training load compared with the general population. We therefore also compared our incidences of osteoarthritis to the rates in the general population by data (generous support from Professor Gunnar Engström) from a large Swedish population-based cohort study well representing the distribution of body mass index, the prevalence of smoking and sociodemographic status and the average incidence of hip and knee joint replacement in Sweden [23]. With an identical definition of knee- and hip osteoarthritis in the studies, a similar age distribution, calendar period of observation and length of follow-up, the Mantel-Haenszel age- and sex-adjusted incidence rate ratio (IRR) of knee osteoarthritis among participants in Vasaloppet was 1.00, 95% CI 0.80 to 1.25 compared to the general population. The rate of hip osteoarthritis was, however, higher in our cohort (IRR 1.65, 95% CI 1.37 to 1.97), which indicates a conservative estimation of the risk for hip osteoarthritis by physical activity in the present study. Nevertheless, compelling evidence exists that a moderate and high VO_2_max and physical activity reduce the risk of numerous chronic diseases and provide higher longevity [1], [3]. There seems, however, only to be small additional benefits on longevity by vigorous exercise compared with moderate intensity training [1], [3], [37]. Our study suggests that intensive exercise might even harm joint cartilage. At the same time, restrained exercise may be a protective factor and is generally recommended for the patient with osteoarthritis [15]. In fact, moderate levels of exercise can even exert small short-time benefits of pain relief among those with osteoarthritis [38].

Major advantages with our design were the large study size, the individual personal registration number provided to all Swedish citizens that enabled linkage between registers with virtually no loss at follow-up and no missing data, and the use of the combination of a diagnostic code for primary osteoarthritis with a surgical procedure code as a measure of a major clinical outcome. We were able to adjust our associations for injuries and exclude those with prevalent osteoarthritis. Our exposures are objective and they are closely related to, albeit proxy measures of, training intensity and duration [39]. Elite skiers do not only perform cross-country skiing exercises but also frequently long-distance running and roller skiing [39], a fact that only conservatively biases our estimates since previous injuries from any sports were identified by the register data.

Our study also has possible limitations. We did not have information on anthropometric measures, and excess body weight increases the risk of both hip and knee osteoarthritis [23]. Nevertheless, participants with many successful races combined with a fast race performance were those who were most likely to have a future diagnosis of severe osteoarthritis: these participants are not likely to have a higher body mass index than participants with low skiing speed. Our inclusion period and exposure assessment was limited to the period 1989 through 1998. Information regarding ski races before 1989 is lacking. The consequence of this conceivable misclassification of our exposures would tend to bias the results toward the null hypothesis. Most participants, however, had their last race during the later part of the inclusion period and this is also illustrated by our supplemental analysis when restricting the cohort to skiers with latest race participation in either of the two last years of the inclusion period. The effect was marginal on our estimates. By our register data, we might have overlooked mild cartilage injuries that might eventually have led to osteoarthritis. Such mild injuries are more likely to have occurred in the knee joint than in the more constrained hip joint [40], and in fact, the higher risk of hip osteoarthritis after intensive exercise in our study was not lower than that for knee osteoarthritis. Moreover, we found no significant evidence of higher frequency of registry reported injuries among participants with many races and a fast skiing speed compared to those with only one slow pace race. Indication bias is a further conceivable limitation if elite athletes had more easy access to health care. Surgery for severe osteoarthritis, however, is not unequally provided in the Swedish tax funded public health care system. The few privately funded operations done in Sweden are not reported to the National Patient register. Theoretically, genetic predisposition to both high physical performance capacity and an increased risk of osteoarthritis might be an underlying mechanism explaining our results. Indeed, both osteoarthritis [41] and exercise level [42] cluster in families and have a modest heritability [41], [42]. Nevertheless, linkage and whole-genome analyses have not revealed chromosome sites or individual gene variants in common for osteoarthritis and athletic behaviour [41], [43].

We conclude that intensive exercise, at a level required to complete multiple and fast long-distance skiing races, is associated with a higher risk of severe knee and hip osteoarthritis.

## Supporting Information

Table S1 Distribution of participants and number of cases with incident severe osteoarthritis of the hip and knee during follow-up by year of the last race.
(DOC)
