# Supplementary material for: Risk of Severe Knee and Hip Osteoarthritis in Relation to Level of Physical Exercise: A Prospective Cohort Study of Long-Distance Skiers in Sweden
Source: PLoS One. 2011 Mar 30;6(3):e18339. doi: 10.1371/journal.pone.0018339 (PMC3068188; doi:10.1371/journal.pone.0018339)
Supplement: Table S1 — Distribution of participants and number of cases with incident severe osteoarthritis of the hip and knee during follow-up by year of the last race. (DOC) [file pone.0018339.s001.doc]

**Table S1.** Distribution of participants and number of cases with incident severe osteoarthritis of the hip and knee during follow-up by year of the last race

| **Year of last race** | **N participants** | **% of total number of participants** | **N with osteoarthritis** | **% of osteoarthritis cases** |
| --- | --- | --- | --- | --- |
| **1989** | 1334 | 2.5 | 16 | 2.8 |
| **1991** | 3039 | 5.6 | 58 | 10.2 |
| **1992** | 2145 | 4.0 | 24 | 4.2 |
| **1993** | 3032 | 5.6 | 48 | 8.4 |
| **1994** | 5048 | 9.4 | 56 | 9.8 |
| **1995** | 5201 | 9.6 | 67 | 11.8 |
| **1996** | 6805 | 12.6 | 63 | 11.1 |
| **1997** | 7904 | 14.6 | 61 | 10.7 |
| **1998** | 19475 | 36.1 | 177 | 31.1 |
| **Total** | 53983 | 100 | 570 | 100 |
